# Earth-vertical motion perception assessment using an elevator: a feasibility study

**DOI:** 10.1038/s41598-023-36655-7

**Published:** 2023-06-09

**Authors:** Simona Schellenberg, Dominik Straumann, David Andrew Green, Philipp Schuetz, Yves Zehnder, Jaap Swanenburg

**Affiliations:** 1 Physiotherapy and Occupational Therapy Research Center, Directorate of Research and Education, University Hospital Zurich, Zurich, Switzerland; 2 Department of Neurology, University Hospital and University of Zurich, Zurich, Switzerland; 3 Clinical Neuroscience Center, University Hospital Zurich, Zurich, Switzerland; 4 Space Medicine Team, HRE-OM, European Astronaut Centre, European Space Agency, Cologne, Germany; 5 KBRwyle Laboratories GmbH, Cologne, Germany; 6 Centre of Human & Applied Physiological Sciences, King’s College London, London, UK; 7 Lucerne University of Applied Sciences and Arts, Lucerne, Switzerland; 8 Institute of Anatomy, Faculty of Medicine, University of Zurich, Winterthurerstrasse 190, 8057 Zurich, Switzerland; 9 Air Force Center, Air Base Dübendorf, UZH Space Hub, Zurich, Switzerland

**Keywords:** Neurological disorders, Sensory systems

## Abstract

A feasible, inexpensive, rapid, and easy-to-use method to measure vestibular vertical movement perception is needed to assess the sacculus-mediated low-frequency otolith function of dizzy patients. To evaluate the feasibility of reaction time assessment in response to vertical motion induced by an elevator in healthy young individuals. We recorded linear acceleration/deceleration reaction times (LA-RT/LD-RT) of 20 healthy (13 female) subjects (mean age: 22 years ± 1 SD) as a measure of vertical vestibular motion perception. LA-RT/LD-RT were defined as the time elapsed from the start of elevator acceleration or deceleration to the time at which subjects in a sitting position indicated perceiving a change in velocity by pushing a button with their thumb. The light reaction time was measured as a reference. All 20 subjects tolerated the assessment with repeated elevator rides and reported no adverse events. Over all experiments, one upward and four downward rides had to be excluded for technical reasons (2.5%). The fraction of premature button presses varied among the four conditions, possibly related to elevator vibration (upward rides: LA-RT-up 66%, LD-RT-up 0%; downward rides: LA-RT-down 12%, LD-RT-down 4%). Thus LD-RT-up yielded the most robust results. The reaction time to earth-vertical deceleration elicited by an elevator provides a consistent indicator of linear vestibular motion perception in healthy humans. The testing procedure is inexpensive and easy to use. Deceleration on upward rides yielded the most robust measurements.

## Introduction

The perception of one’s own head and/or body movement in space is critical for gait, posture control, and spatial orientation^1^. This self-motion perception results from the integration of vestibular, visual, and somatosensory information^2^. Among the sensors in the inner ear, the otolith organs—the sacculus and the utriculus—provide the necessary signal for the perception of linear acceleration or tilt of the head relative to gravity^3^. Due to its orientation within the skull, the sacculus is more sensitive to earth-vertical linear translation (e.g., elevator acceleration) when the head is upright, whereas the utriculus is more sensitive to horizontal linear translation (e.g., car acceleration)^4^.

The most widely used auxiliary test of otolith function used by neuro-otologists are vestibular-evoked myogenic potentials (VEMPs)^5,6^. VEMPs, however, have several limitations^7^, including being technically complex, time-consuming, and relatively expensive. Moreover, VEMPs demonstrate significant response variabilities that depend on non-vestibular factors, such as hearing acuity, skull thickness, and the degree of tonic muscle activation^7^. While ocular VEMPs (oVEMPs) reflect utricular high-frequency function, low-frequency utricular function is typically assessed with subjective visual vertical (SVV) test^8^. In routine saccular assessment, however, only high-frequency function is measured using oVEMPs. In other words, a low-frequency sacculus-mediated equivalent to the mainly utriculus-mediated SVV is missing. Thus, a simple, easy to employ, and relatively low-cost method to assess low-frequency vertical otolith function could be a valuable element of the vestibular testing battery for evaluating dizzy patients.

As gravity is equivalent to a constant earth-vertical acceleration, an earth-vertical body movement only increases (upward acceleration or downward deceleration) or decreases (downward acceleration or upward deceleration) the length of the gravito-inertial (GI) vector, but not its direction. With subjects in the upright position, sensing GI-vector length changes by the otolith organs relies predominantly on the function of the saccular maculae due to their orientation relative to the skull^9^. As is the case for any vestibular stimulus, other sensory inputs, such as vision, proprioception, and hearing, contribute to the perception of linear motion. Thus, disorders of saccular pathways and pathways of other involved sensory inputs may increase the threshold of vertical linear motion perception^10^.

Motion platforms has been successfully employed utilized to provide earth-vertical motion stimuli; they are, however, technologically complex and expensive and therefore their clinical application is rare^11–13^. A suitable elevator, such as one used in hospitals for the transport of beds, could provide a simple, easy to employ, and relatively low-cost alternative to clinically assessing vertical linear motion perception in patients, including subjects with suspected deficits in saccular pathways.

Reaction times are frequently used in auxiliary neurological assessments, e.g., in patients with suspected neurocognitive impairment or concussion^14,15^. Reaction times could also permit the evaluation of linear self-motion perception and thus provide a valuable vestibular screening tool for the detection of otolith dysfunction, such as in patients with chronic peripheral vestibular hypofunction^16^ or with otolith hypersensitivity caused by vestibular migraine^17^.

To our knowledge, no studies have attempted to assess vestibular perception via reaction time to elevator-induced vertical acceleration and deceleration. This study evaluated the feasibility of a linear acceleration/deceleration reaction time (LA-RT or LD-RT) assessment in response to low-frequency vertical motion induced by a hospital elevator in healthy young individuals.

## Results

### Feasibility of elevator rides

**Figure 1:**
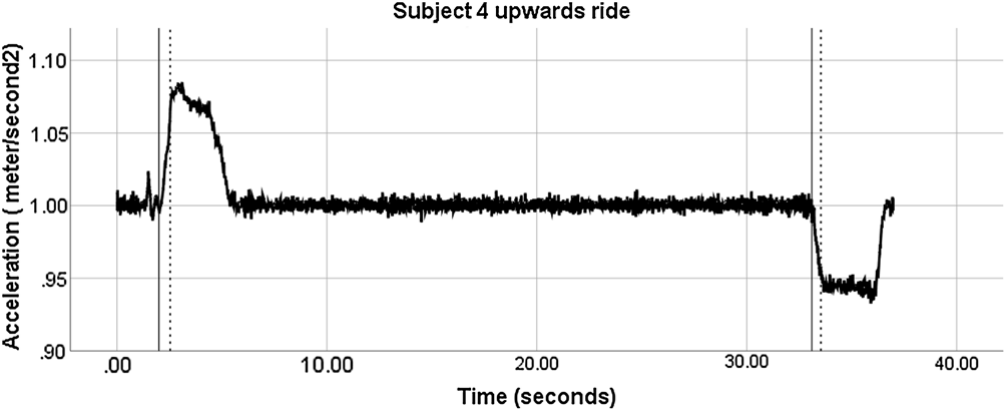
Example of elevator acceleration and deceleration during an upward ride. Solid vertical lines: start of acceleration or deceleration. Dotted line: button press of the participant (subject 4) indicating the perception of vertical motion, i.e., the change of vertical velocity. The reaction times (LA-RT: linear acceleration reaction time; LD-RT: linear deceleration reaction time) are defined by the interval between the solid and the dotted lines.

There were no adverse events such as dizziness or disorientation. All participants confirmed that the assessment procedure was tolerable and acceptable for being repeated several times. In every subject, the testing, including preparation, could be performed in less than 20 min. One upward ride and four downward rides were excluded for technical reasons (2.5%). The acceleration curves of 25 upward and 25 downward elevator rides showed excellent reliability: curves of upward had an ICC score of 0.986 (CI 0.986–0.987) and a Cronbach’s alpha of 0.986, while curves of downward had an ICC score of 0.990 (CI 0.990–0.991) and a Cronbach’s alpha of 0.990. A typical acceleration curve during an upward elevator ride is shown on Fig. 1.

### Feasibility of reaction time measurements

After removing outliers, normality tests showed no normal distribution of reactions times upon elevator linear accelerations (LA-RT) and elevator linear decelerations (LD-RT). Boxplots of all LA-RT and LD-RT raw data with outliers can be found in Fig. S1 of the supplementary file. The data distribution of the LA-RT and LD-RT of the upward and downward elevator rides is given in the Table 1.

**Table 1:** Linear acceleration and deceleration reaction time.

	Upward rides		Downward rides	
	LA-RT	LD-RT	LA-RT	LD-RT
Mean; seconds	− 0.07	0.45	0.19	0.40
Standard error of mean	0.04	0.02	0.03	0.02
Median; seconds	− 0.24	0.47	0.26	0.43
Mode; seconds	− 0.90	0.44	− 0.82	0.60
Standard deviation	0.44	0.15	0.25	0.19
Variance	0.19	0.02	0.06	0.04
Range; seconds	1.64	0.79	1.42	0.77
Minimum; seconds	− 0.90	0.09	− 0.82	− 0.07
Maximum; seconds	0.74	0.87	0.60	0.70
Outlier; count	5	1	5	4
Premature button press; count (% of total)	55 (66%)	0 (0%)	11 (12%)	3 (3%)
Acceleration at button press; m/s^2^ (Standard deviation)	0.29 (0.23)	− 0.37 (0.12)	− 0.29 (0.19)	0.28 (0.14)
Light reaction time correlation; r (p = significant)	0.181 (p = 0.132)	0.057 (p = 0.811)	0.045 (p = 0.850)	0.074 (p = 0.758)

*LA-RT* linear acceleration reaction time, *LD-RT* linear deceleration reaction time.

**Figure 2:**
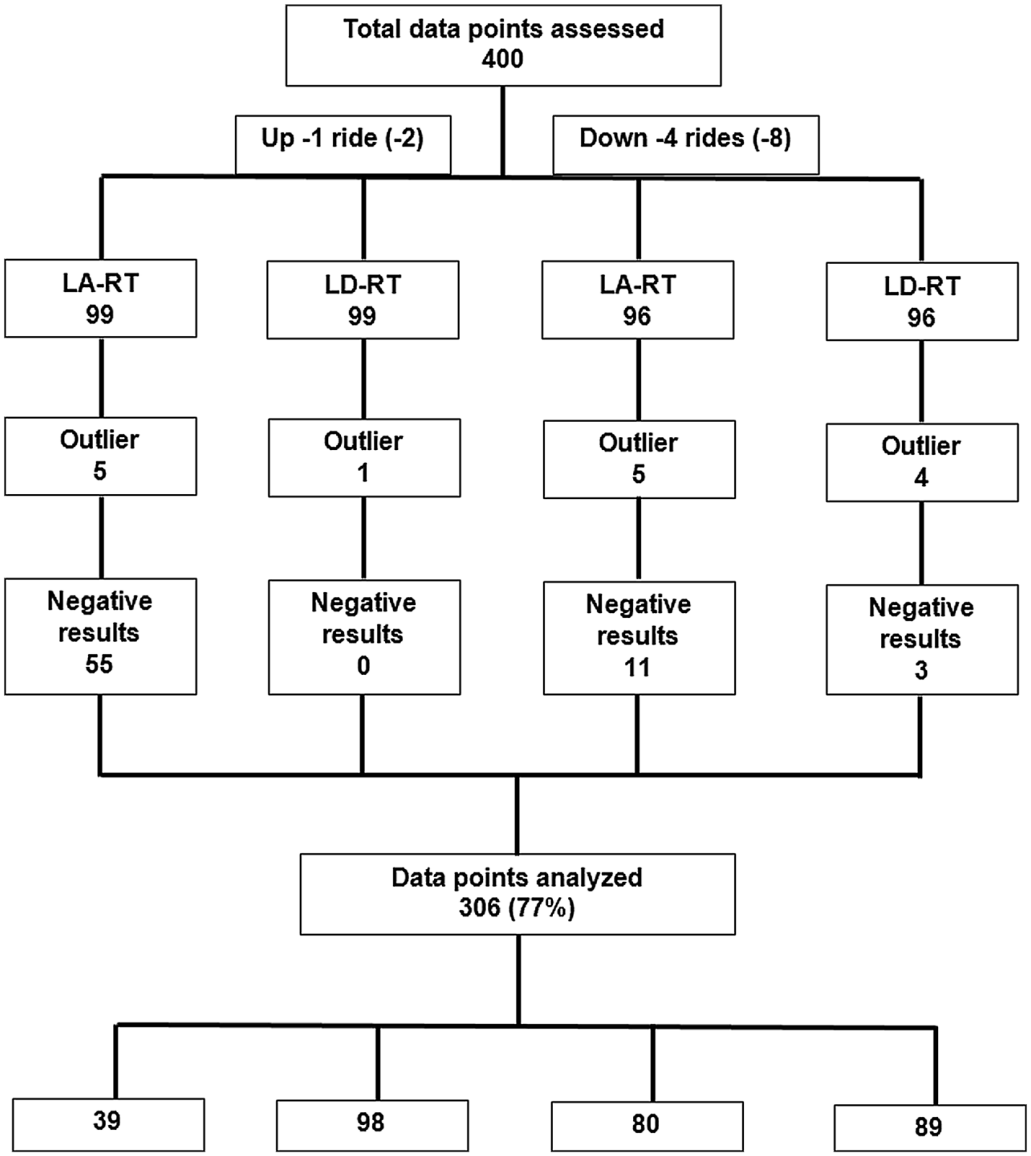
Flowchart of data analysis. ‘Data points’ refer to periods of elevator acceleration or deceleration over all subjects tested.

Negative reaction times (premature button press) are reactions that occurred before the actual start of acceleration or deceleration (i.e., too early). Positive reaction times occurred after the actual onset of acceleration or deceleration (i.e., they likely reflected the reaction to the elevator motion). The flowchart of data analysis is shown in Fig. 2.

**Figure 3:**
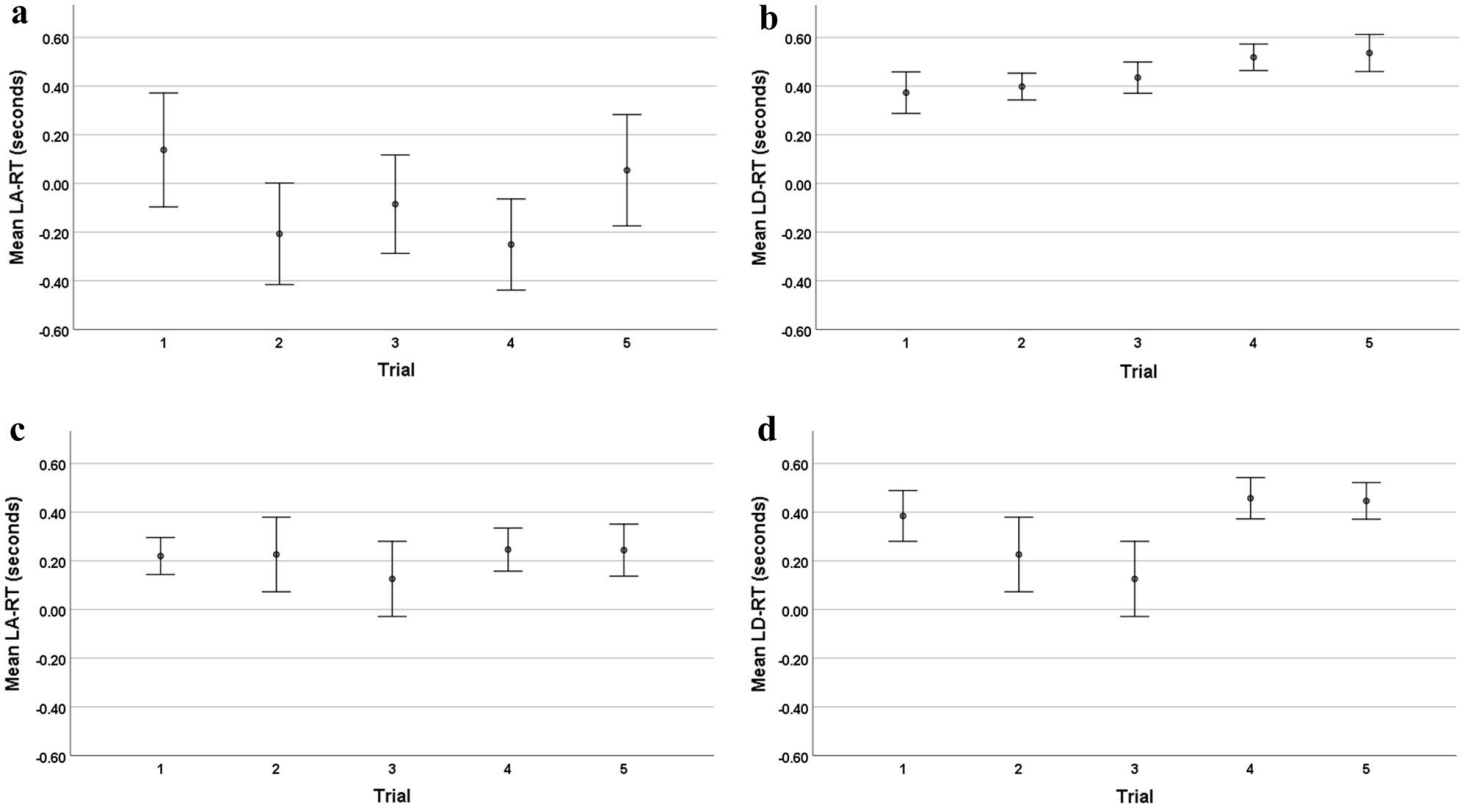
Mean reaction times ± 1 SE of sequential 5 trials over all subjects. *LA-RT* linear acceleration reaction time. *LD-RT* linear deceleration reaction time. (**a**) Acceleration during upward elevator rides. (**b**) Deceleration during upward acceleration rides. (**c**) Acceleration during downward elevator rides. (**d**) Deceleration during downward acceleration rides.

The five upward rides showed a significant change with ride repetition in LA-RT-up (x^2^ = 13.40, df = 4, p = 0.009) and a significant effect of LD-RT-up (x^2^ = 16.58, df = 4, p = 0.002). The five downward rides showed no effect on LA-RT-down (x^2^ = 6.33, df = 4, p = 0.176) and significant effect of LD-RT-down (x^2^ = 12.33, df = 4, p = 0.0.015). The mean reaction in all five rides and two direction times are shown in Fig. 3a–d.

## Discussion

During all rides, the participants performed the tasks of indicating elevator motion, i.e., change of elevator velocity, without any difficulties. All participants confirmed the tolerability and acceptability of the repeated assessment, and the elevator rides showed excellent reliability. The vibration of the elevator at the start of the rides probably caused many premature button presses by the participants. The measures of deceleration during the upward elevator rides, however, yielded consistently valid reaction times with the least variability. This is probably related to the fact that the least noisy change in gravito-inertial acceleration (i.e., jerk) was detected during deceleration of the upward moving elevator.

**Figure 4:**
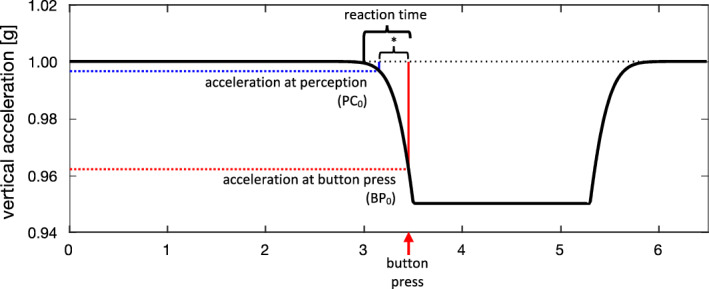
Schematic explanation how earth-vertical motion perception on an elevator is assessed by reaction time. Example of a decelerating elevator at the end of an upward ride. Acceleration (in g) of the elevator (black line) is plotted as a function of time. Deceleration from baseline (1 g) begins at 3.0 s (indicated by small black vertical line) and quickly reaches a plateau at 0.995 g (compare to actual data at the end of an upward ride on Fig. 1). The subject indicates the perception of a changing acceleration by pressing a button (red arrow) 450 ms after the beginning of deceleration. Note that the acceleration value at the moment of button press (red dotted line, 0.0377 g = 0.370 m/s^2^, average value in our population of healthy human subjects) does not represent the acceleration threshold. Rather the threshold was crossed 299 ms (small blue vertical line, average light reaction in our population) earlier. At this time, the deceleration from baseline (blue dotted line, 0.00323 g = 0.0317 m/s^2^, theoretical value) was approximately 10 times lower than at button press.

Since the change in acceleration during elevator rides is slow and thus provides a low-frequency stimulus (half a period is around three seconds, or 0.16 Hz), subjects may have difficulty to distinguish derivative of elevator acceleration or deceleration (jerk) from noise due to motor vibration. We hypothesize that the reaction time upon elevator acceleration or deceleration (Fig. 4, reaction time) is composed of two parts: (1) The time that elapses from the beginning of elevator acceleration or deceleration to the moment when the perceptual threshold is reached; (2) the time that elapses between reaching the threshold and the button press. Conceivably, the second part (Fig. 4, asterixs) is equivalent to the light reaction time. The acceleration value at the time of the button press was in the range of the perception threshold found in a study using a two-interval forced-choice task when we take the light reaction time for the time elapsing between motion perception and button press (see hypothetical values in the legend of Fig. 4)^18^. Since we are not able to vary the acceleration and deceleration parameters of the elevator, our hypothesis can only be tested by measuring subjects with increased perceptual thresholds, i.e., patients with deficits of the sacculi or their afferents.

Over the course of the five elevator rides, the LD-RT significantly increased. This could be the result of habituation^19^ as well as fatigue. Habituation is an increase in reaction that can frequently be observed with repeated sensory stimulation^20^. Since other confounding sensory inputs were minimised and the motor output (button press) remained the same, the increase in LD-RT may have reflected vestibular threshold habituation.

An important aspect not investigated in this study was whether the vibrations of the elevator were transmitted to the chair during the accelerations. Furthermore, elevators may, of course, have different motion characteristics. Therefore, it is important to evaluate the magnitude and repeatability of acceleration/deceleration profiles generated by each individual elevator. One must note that, unlike cervical vestibular-evoked myogenic potentials (cVEMPS), an elevator test—as all vertical motion devices—cannot assess unilateral saccular function. Another limitation of our study may be that the inertial measurement unit (IMU) was placed on the floor of the elevator. Therefore, the potential confounding movement of the head was not assessed. However, subjects were instructed to keep their heads still to maximise saccular activation and to minimise any extra-saccular activation. Another weakness of the elevator procedure is that it is possible that the exact threshold is not measurable, as the velocity of the elevator may change too quickly. An ongoing study is addressing whether the acceleration/deceleration of the elevator is indeed too fast for vestibular patients with increased thresholds.

In a recently published study, the otolith system’s ability to perceive self-motion was tested by investigating adaptive responses to asymmetric off-axis vertical rotation. The results revealed that after conditioning in the upright position, adaptive perceptual errors increased by 50%^21^. This raises the possibility of further perceptual errors that should be explored in subsequent studies.

Our approach to saccular stimulation is inexpensive, not time consuming, and easy to apply, with little requirement for operator training^22^. Indeed, for clinical purposes, rapid—even if imperfect—can greatly assist clinical diagnosis without complex and time-consuming motion platform-based assessment^11,12^. Deceleration of the elevator (upward) yielded the most valid measurements, as jerk motion was minimal. In addition, short-duration assessment is advantageous to minimise the risk of vertigo or dizziness and to minimise any effect of attention deficits, which have been reported in vestibular patients^12^.

## Conclusion

The reaction time to earth-vertical deceleration elicited by an elevator provides a consistent indicator of linear vestibular motion perception in healthy humans. The testing procedure is inexpensive and easy to use. Deceleration of the elevator during upward rides yielded the most robust measurements, with no premature indication of perceived deceleration by the subjects. Further evaluation of repeatability and threshold responses is warranted, in addition to comparison with the responses of patients with relevant vestibular dysfunctions.

## Methods

### Participants

Twenty (7 male) healthy young adults aged between 18 and 30 years (mean age 22.6 ± 1.2 years; mean height: 176.4 ± 11.2 cm; mean weight: 67.1 ± 12.4 kg; mean BMI: 21.4 ± 2 kg/m2) were recruited having provided written informed consent to participate in the study approved by the ethics committee of the Canton of Zurich under BASEC 2019-01759 and registered at ClinicalTrials.gov Identifier: NCT04200820 at 16/12/2019. Testing was carried out in accordance with relevant guidelines and regulations. The exclusion criteria were as follows: (1) acute pain, (2) chronic neck pain, (3) previous neck surgery, (4) any vestibular disorder, (5) fear of elevators or claustrophobia, (6) gait disorders putatively attributed to causes other than vestibular dysfunction, and (7) a score of > 30 on the dizziness handicap inventory (DHI). The DHI is a validated, 25-item, self-reported questionnaire used to evaluate the self-perceived handicapping effects caused by dizziness^23^. The higher the score, the greater the degree of handicap. All testing was performed at the Department of Neurology, University Hospital Zurich, Switzerland.

### Study procedures

Participants visited the department on a single occasion for approximately 30 min. Each participant was exposed to five upward and five downward elevator rides. Linear acceleration reaction times (LA-RT) were recorded on the upward (A-up) and downward (A-down) accelerations, and linear deceleration reaction times (LD-RT) were recorded on the upward (D-up) and downward ride decelerations (D-down). Following completion of the elevator rides, participants were asked if they had experienced any adverse events or symptoms.

**Figure 5:**
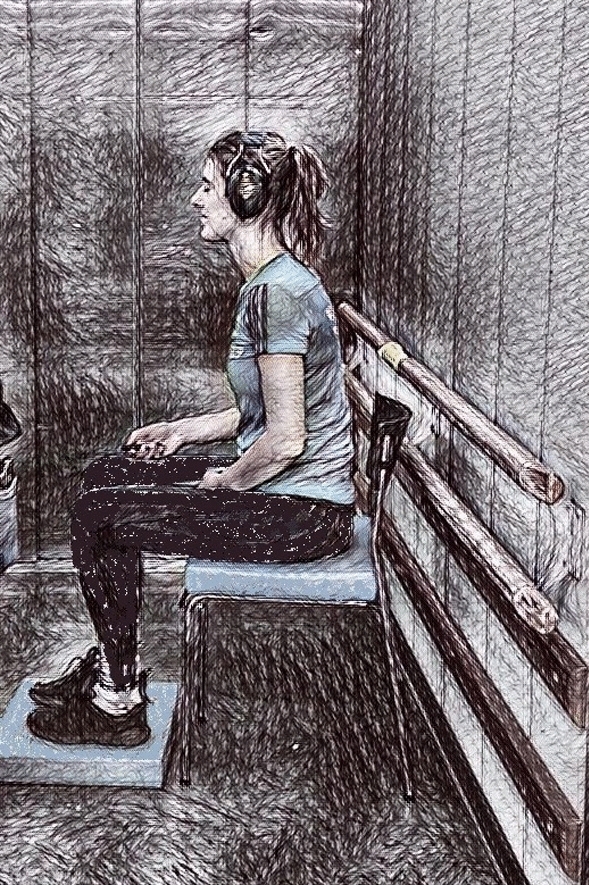
Experimental setup. The subject is sitting with eyes closed on a foam block (blue) wearing a noise-cancelling headset. The remote control for button press is operated with one hand.

Prior to the lift rides, the light reaction time was recorded. During the lift ride, the participants were asked to close their eyes and wear a (white) noise-cancelling headset. To minimise lower limb proprioceptive sensory information, participants were seated on foam, and their feet were placed on foam blocks (Airex balance pads, 3A Composites Holding AG, Switzerland)^24^ (Fig. 5). The vestibular vertical movement perception measurements were performed in a 16-floor bed elevator in a hospital building (Nord1, University Hospital Zurich, Switzerland). Data collection was instigated prior to the beginning of the elevator ride and terminated once the elevator had made a full stop on the respective floors (1 and 16). The acceleration and deceleration duration was 3.15 s, with a peak acceleration of 0.07 m/s^2^.

Elevator rides are clearly not optimal for inducing linear acceleration or deceleration due to accompanying vibrations at the start and end of the elevator motion. Therefore, the reliability of the acceleration curve of elevator rides and the variability of acceleration or deceleration of the bevor at the start and end were tested to determine which of the measurements was feasible. This study followed the STROBE checklist.

### Linear velocity reaction time (LVRT)

**Figure 6:**
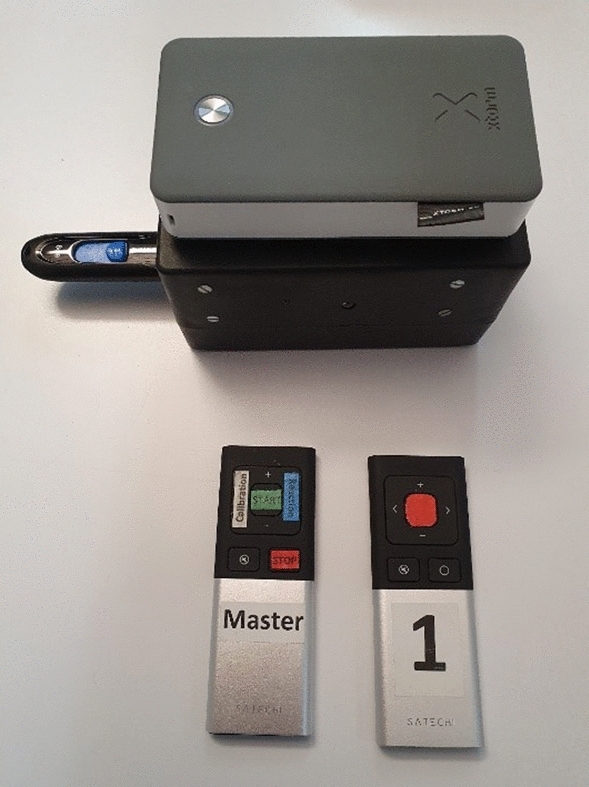
Recording device containing the sensor, the battery (gray) and an USB stick for data stortage; remote for the experimenter (Master); remote control for subject (1).

LVRT was recorded via an inertial measurement unit (IMU) (FXOS8700 CQ from NXP mounted on an adafruit (“NXP Precision 9DoF” Breakout board) to record vertical acceleration connected to two Bluetooth remote controls (Satechi ST-ARCM) (Fig. 5). The master remote control was operated by the experimenter, instigating and terminating the recording. The second remote control was used by the log the state of the participant’s trigger button (red), which they pressed with their thumb when they sensed motion (Fig. 6). A Raspberry Pi CPU was used to record linear acceleration and trigger/controller status.

### Acceleration/deceleration reaction time (LA-RT or LD-RT)

**Figure 7:**
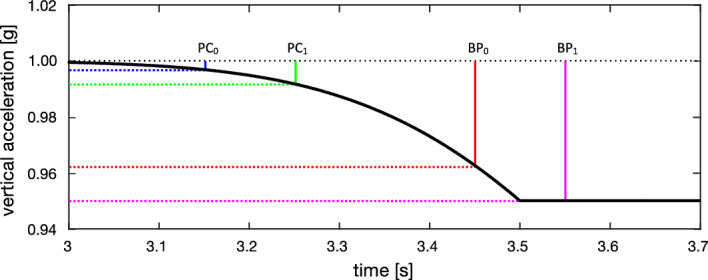
Same trajectory of elevator deceleration as in Fig. 4, but with enlargement along the x-axis (interval: 3.0 to 3.7 s). PC_0_ indicates the moment of motion perception and BPo the moment of button press in a healthy subject. Assume that a patient with elevated threshold for earth-vertical motion presses the button 100 ms later than normal (BP_1_). In this case, the elevator had already reached the plateau of deceleration from baseline (magenta dotted line, 0.05 g = 0.491 m/s^2^). If the reaction time relative to the moment of motion perception (PC1) is unchanged, i.e., remains at 299 ms, the threshold of deceleration relative to baseline is approximately 2.5 times higher than in healthy subjects (green dotted line, 0.0083 g = 0.0819 m/s^2^).

LA-RT or LD-RT was defined as the time elapsed from the start of the elevator acceleration or deceleration to the time that the subjects indicated a perception of a change of velocity by pressing their button. Figure 7 explains how LD-RT would be become longer as the perception threshold increased due to saccular deficits. The same mechanism would also apply to vestibular habituation. Future studies should investigate whether further habituation occurs and at what point responses plateau.

### Light reaction time test (LRTT)

LRTT is defined as the time that elapses from the random switch-off of a blue LED until the participant reacts by pressing the red button with the thumb as quickly as possible^25^. After a trial run, the test was performed twice and averaged. The literature reports that LRTT is typically shorter than LA-RT/LD-RT^26^. For confirmation, LRTT was compared with the LA-RT/LD-RT results.

### Data processing

LVRT data (150 Hz) were processed with a script in Python 3 (Python Software Foundation, http://www.python.org) using the Pandas Package. To reduce the impact of shot noise, an average running filter with a 300 ms window was applied. Subsequently, the acceleration and declaration peaks were identified using the find_peak algorithm from the scipy signal package. The start and end times of accelerations and decelerations were defined as the time points when the denoised signal reached 95% of its peak value, respectively. The time points of the participants’ reaction times were identified by the button press with respect to the start/end times.

### Statistical analysis

#### Feasibility elevator

The reliability of the acceleration curve of elevator rides (upward and downward) was assessed with an intraclass correlation coefficient (ICC) with a 95% CI. Cronbach’s alpha was assessed to evaluate internal consistency.

#### Feasibility LA-RT and LD-RT

Data normality was tested using the Shapiro–Wilk test after removing outliers (× 3 interquartile range). The effect of trial repetition (N = five trials) was evaluated using the Friedman test, with a significance level of α = 0.05. Two LRTTs were performed; the first two LA-RT/LD-RT measurements were used for comparison. Correlation of mean was assessed via Spearman testing. Correlation coefficients were interpreted as follows: r < 0.50, weak relationship; 0.50 ≤ r ≤ 0.74, moderate relationship; and r ≥ 0.75, strong relationship^27^. All statistics were performed with SPSS Statistics 25 for Windows (IBM Inc., USA).

## Supplementary Information

Supplementary Figure S1.

## Data Availability

The datasets generated during and/or analysed during the current study are available from the corresponding author on reasonable request.
