# Supplementary material for: Earth-vertical motion perception assessment using an elevator: a feasibility study
Source: Sci Rep. 2023 Jun 9;13:9450. doi: 10.1038/s41598-023-36655-7 (PMC10256722; doi:10.1038/s41598-023-36655-7)
Supplement: Supplementary file 1 — Supplementary Figure S1. [file 41598_2023_36655_MOESM1_ESM.docx]

Supplementary Material

**Supplementary** Figure 1a-b

Distribution of ***LA-RT and LD-RT*** elevator rides upwards with outliers

| 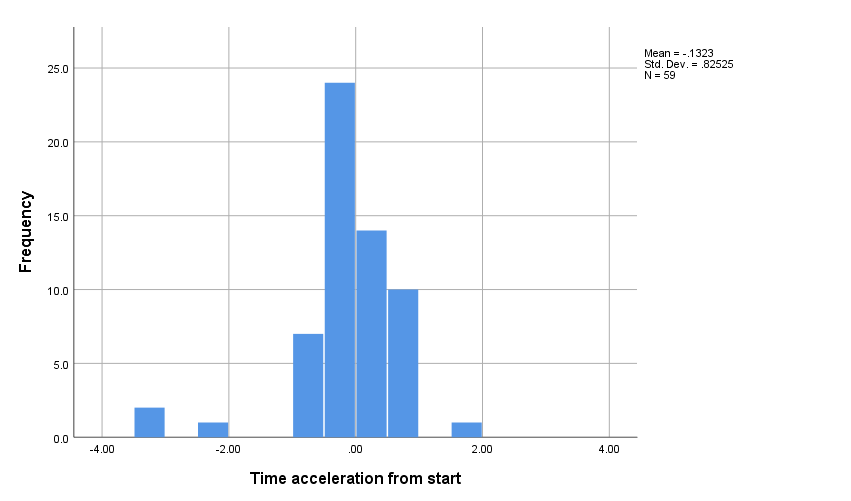 | 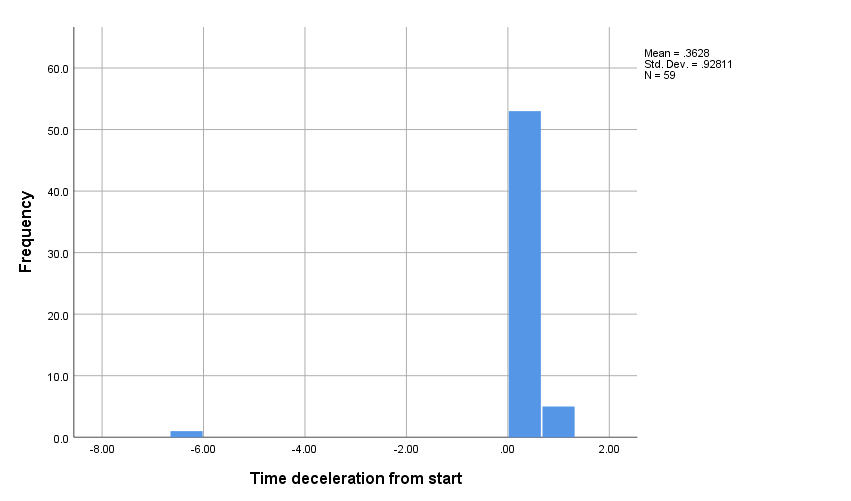 |
| --- | --- |
| 1a LA-RT | 1b LD-RT |

Figure 1c-d

Distribution of ***LA-RT and LD-RT*** elevator ride downwards with outliers

| 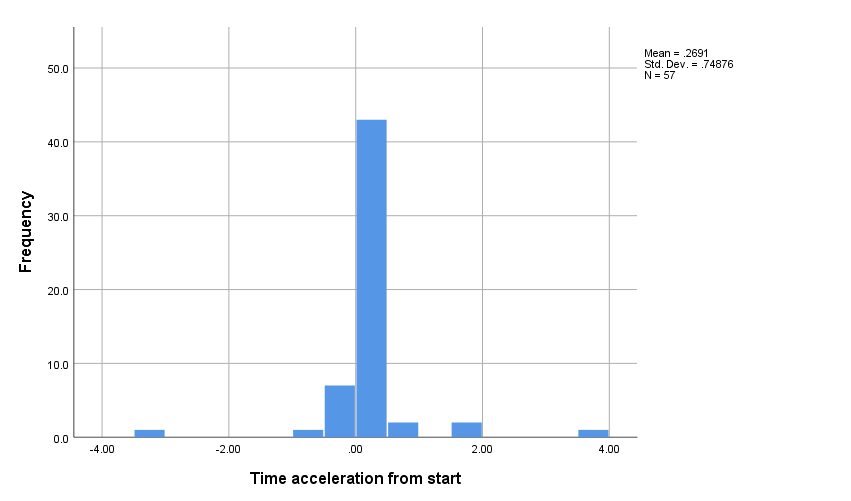 | 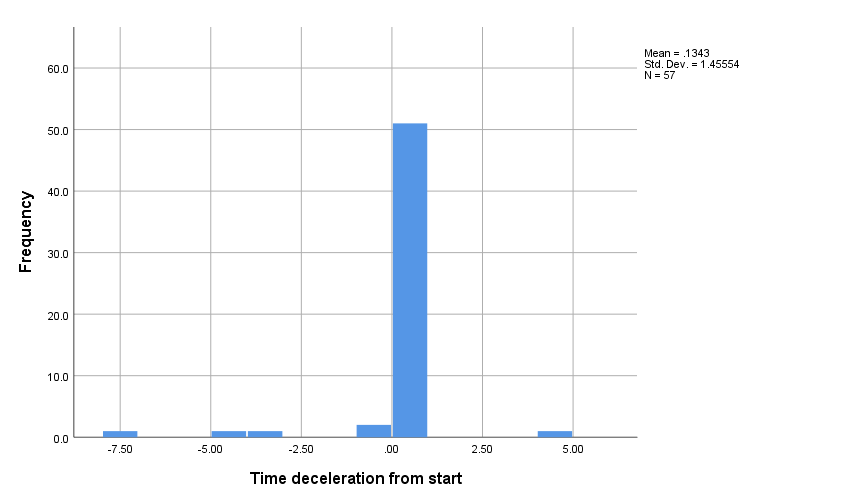 |
| --- | --- |
| 1c LA-RT | 1d LD-RT |
